# Interactions between circulating inflammatory factors and autism spectrum disorder: a bidirectional Mendelian randomization study in European population

**DOI:** 10.3389/fimmu.2024.1370276

**Published:** 2024-04-29

**Authors:** Junzi Long, Hui Dang, Wenlong Su, Md. Moneruzzaman, Hao Zhang

**Affiliations:** ^1^ School of Rehabilitation, Capital Medical University, Beijing, China; ^2^ Department of Neurorehabilitation, China Rehabilitation Research Center, Beijing, China; ^3^ Division of Brain Sciences, Changping Laboratory, Beijing, China; ^4^ Cheeloo College of Medicine, Shandong University, Jinan, Shandong, China

**Keywords:** autism spectrum disorder, inflammatory factors, inflammation, Mendelian randomization, single nucleotide polymorphisms, genome-wide association study

## Abstract

**Background:**

Extensive observational studies have reported an association between inflammatory factors and autism spectrum disorder (ASD), but their causal relationships remain unclear. This study aims to offer deeper insight into causal relationships between circulating inflammatory factors and ASD.

**Methods:**

Two-sample bidirectional Mendelian randomization (MR) analysis method was used in this study. The genetic variation of 91 circulating inflammatory factors was obtained from the genome-wide association study (GWAS) database of European ancestry. The germline GWAS summary data for ASD were also obtained (18,381 ASD cases and 27,969 controls). Single nucleotide polymorphisms robustly associated with the 91 inflammatory factors were used as instrumental variables. The random-effects inverse-variance weighted method was used as the primary analysis, and the Bonferroni correction for multiple comparisons was applied. Sensitivity tests were carried out to assess the validity of the causal relationship.

**Results:**

The forward MR analysis results suggest that levels of sulfotransferase 1A1, natural killer cell receptor 2B4, T-cell surface glycoprotein CD5, Fms-related tyrosine kinase 3 ligand, and tumor necrosis factor-related apoptosis-inducing ligand are positively associated with the occurrence of ASD, while levels of interleukin-7, interleukin-2 receptor subunit beta, and interleukin-2 are inversely associated with the occurrence of ASD. In addition, matrix metalloproteinase-10, caspase 8, tumor necrosis factor-related activation-induced cytokine, and C-C motif chemokine 19 were considered downstream consequences of ASD.

**Conclusion:**

This MR study identified additional inflammatory factors in patients with ASD relative to previous studies, and raised a possibility of ASD-caused immune abnormalities. These identified inflammatory factors may be potential biomarkers of immunologic dysfunction in ASD.

## 1 Introduction

Autism spectrum disorders (ASD) are defined as a group of neurodevelopmental conditions of childhood with environmental causes that are still not fully understood. Most environmental factors during the perinatal stage appear to converge into a series of inflammatory conditions, such as bacterial and viral infections and inflammatory bowel disease (1, 2), suggesting that inflammatory responses could be an underlying factor in the etiology of ASD. Large population-based epidemiological studies have linked ASD with autoimmune disease and abnormal blood levels of various inflammatory cytokines and immunological biomarkers (3, 4). For instance, previous studies on inflammatory biomarkers have found increased concentrations of pro-inflammatory factors IL-1β, IL-4, IL-6, IL-8, and TNF-α (5–8), as well as decreased concentrations of anti-inflammatory factors IL-10 and IL-1Ra (9) in the peripheral blood of patients with ASD. These abnormal inflammatory cytokine levels are linked to greater impairments in language function and social interaction in children with ASD (6, 8). Therefore, it is necessary to further identify inflammatory biomarkers in ASD and uncover the causality between ASD and changes in the levels of inflammatory factors.

Observational studies are often susceptible to confounding, reverse causation, and multiple biases, which can lead to unreliable findings regarding the causal effects of exposures on outcomes. The Mendelian randomization (MR) method provides an alternative approach to investigate causality in epidemiological research, by utilizing genetic variants as instrumental variables to determine whether a risk factor has a causal effect on a health outcome. As an individual’s genotype is determined at conception and cannot be altered, this method avoids the reverse causality between genotype and outcome. In general, MR analysis rests on 3 assumptions: (1) genetic variants are associated with the risk factor; (2) genetic variants are not associated with confounders; and (3) genetic variants affect the outcome only through the risk factor (10). The advent of large-scale genome-wide association studies (GWAS) increases the accessibility of single-nucleotide polymorphisms (SNPs) as instrumental variables to infer causality in MR studies.

Based on the GWAS summary statistics, previous studies have examined causal relationships between 41 circulating inflammatory factors (11) and various complex diseases, including depression (12), epilepsy (13), and Alzheimer’s disease (14). Recently, Zhao et al. (15) extended previous works by conducting a genome-wide protein quantitative trait locus study which identified the genetic architecture of 91 circulating inflammatory factors in 14,824 European-ancestry participants. Several recent studies have included these 91 inflammatory factors for MR analysis, which further extends the use of inflammatory factors in MR studies (16, 17). Although previous observational studies have found a strong association between changes in levels of some circulating inflammatory proteins and ASD (5–9), their causal relationships remain undefined. The number of inflammatory factors analyzed in these studies is also relatively limited. Based on the knowledge above, we conducted the first bidirectional two-sample MR analysis (SNPs associated with the exposure and outcome are individually obtained from two independent samples) to determine the causal relationship between 91 inflammatory factors and ASD. The inflammatory biomarkers identified in this work may provide the basis for an objective test for early and accurate diagnosis of ASD and may shed light on the etiology and pathogenesis of ASD.

## 2 Materials and methods

### 2.1 Study design and data sources

**Figure 1:**
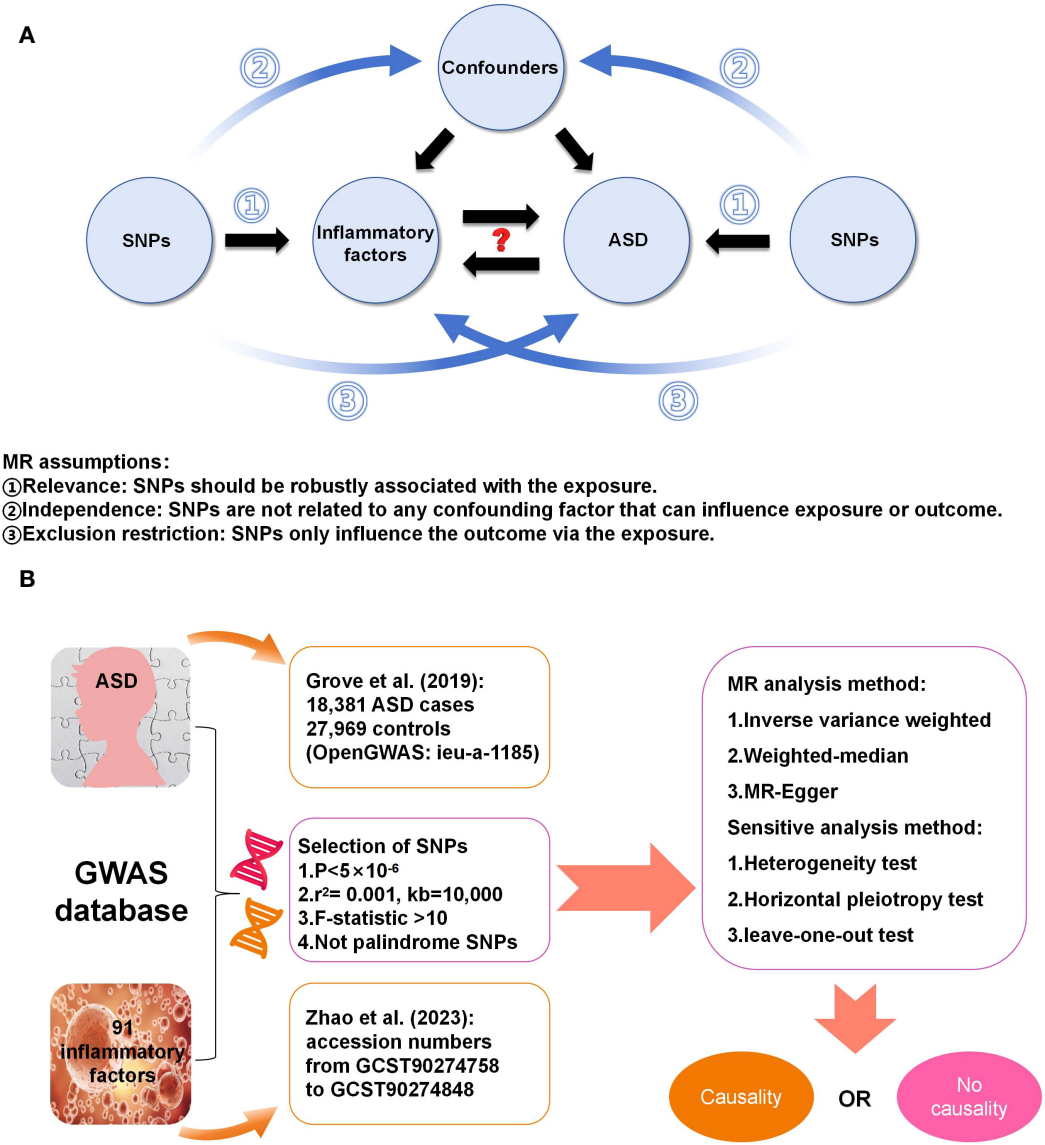
**(A)** Three assumptions in this MR study; **(B)** Workflow of this MR study.

**Figure 1** displays a schematic presentation of the study design. The data were obtained from the GWAS database and all included subjects had provided written informed consent in original research. The GWAS data sets for 91 circulating inflammatory factors are available in the GWAS Catalog (accession numbers from GCST90274758 to GCST90274848). These results come from a recent genome-wide protein quantitative trait locus study of 91 inflammation-related plasma factors in 14,824 European-ancestry participants (15). The genetic data on ASD were obtained from a genome-wide association meta-analysis of 18,381 ASD cases and 27,969 controls by Grove et al. (18) (OpenGWAS: ieu-a-1185). All participants were children born in Denmark between 1981 and 2005, diagnosed with ASD according to the 10th Revision of the International Classification of Diseases before 2014.

### 2.2 Instrument selection

The SNPs strongly associated with inflammatory factors (p < 5e–06) were selected as instrumental variables. The linkage disequilibrium of these SNPs was removed using the clumping procedure in PLINK software (version v1.90), with the linkage disequilibrium parameter (r^2^) < 0.001 and a distance threshold of 10,000 kilobases. The r^2^ was calculated based on the 1000 Genomes Projects reference panel (Genomes Project C et al., 2012) (19). Additionally, we excluded palindrome SNPs and weak instrumental SNPs (F-statistics < 10). The F statistic was calculated by the following equation: F = R^2^ × (N−k−1)/k × (1 − R^2^), where N is the sample size of the exposure factor, K is the number of instrumental variables, and R^2^ is the proportion of variance explained by each instrumental variable. An F-statistic >10 typically indicates a strong correlation between instrumental variables and exposure factors (20).

### 2.3 Statistical analysis

Both statistical and sensitive analyses were conducted using the R software (version 4.2.1) and the “TwoSampleMR” R package (21, 22). The random-effects inverse variance weighted (IVW) method was employed as the main MR analysis to estimate causal effects, complemented by the weighted-median (WM) and MR-Egger methods to investigate potential pleiotropic effects. The IVW method can analyze individual Wald-type ratios of causal effects for each SNP, which provides the most accurate and unbiased causal estimates in the absence of horizontal pleiotropy (23). In the presence of horizontal pleiotropy, the WM method provides a consistent estimate even though half of the genetic variants are invalid instrumental variables (24). The estimation of causal effects of modifiable phenotypes on an outcome relies on the assumption of no pleiotropy, wherein genes solely influence the outcome via the given phenotype. If the genetic variants have a pleiotropic effect on the outcome, then the causal estimates may be biased. The MR-Egger regression intercept test was used to assess residual horizontal pleiotropy – intercepts around the zero indicate that SNPs do not have a direct effect on the outcome via the exposure (25). The Cochran’s Q statistic was utilized to evaluate the heterogeneity of SNPs in both the IVW and MR Egger methods (21). The core assumption of MR is not contradicted even if there is significant heterogeneity in the instrumental variables. A p-value of less than 0.05 suggests the presence of horizontal pleiotropy and heterogeneity. The association of individual SNPs was determined using leave-one-out sensitivity analysis to investigate whether the results were driven by any single SNP (26). For binary exposures, causal estimates were presented as odds ratio (OR) and 95% confidence interval (CI) per log-odds increment of genetic exposure risk. To account for multiple testing, a p-value of 0.00055 (0.05/91) was considered robust significance after the Bonferroni correction. A p-value between 0.00055 and 0.05 was deemed as suggestive evidence of potential causation. All statistical analyses were two-sided.

## 3 Results

### 3.1 Basic information about instrumental variables and exposures

After the selection of SNPs based on the criteria, a total of 1,815 SNPs related to 91 circulating inflammatory factors extracted from the GWAS database were used as instrumental variables (**Supplementary Table S1**). The basic information on 91 circulating inflammatory factors is summarized in **Supplementary Table S2**. Due to the large number of inflammatory factors and associated SNPs, in this section, we focus on the presentation of positive results in MR analysis. SNPs used in the positive results of both forward and reverse MR analysis are shown in **Supplementary Tables S3, S4**. This compilation includes information on chromosome location, effect allele, and effect allele frequency. Moreover, all SNPs had F statistics greater than 10, indicating that they were free of weak instrumental bias.

### 3.2 The causative impact of circulating inflammatory factors on ASD

**Figure 2:**
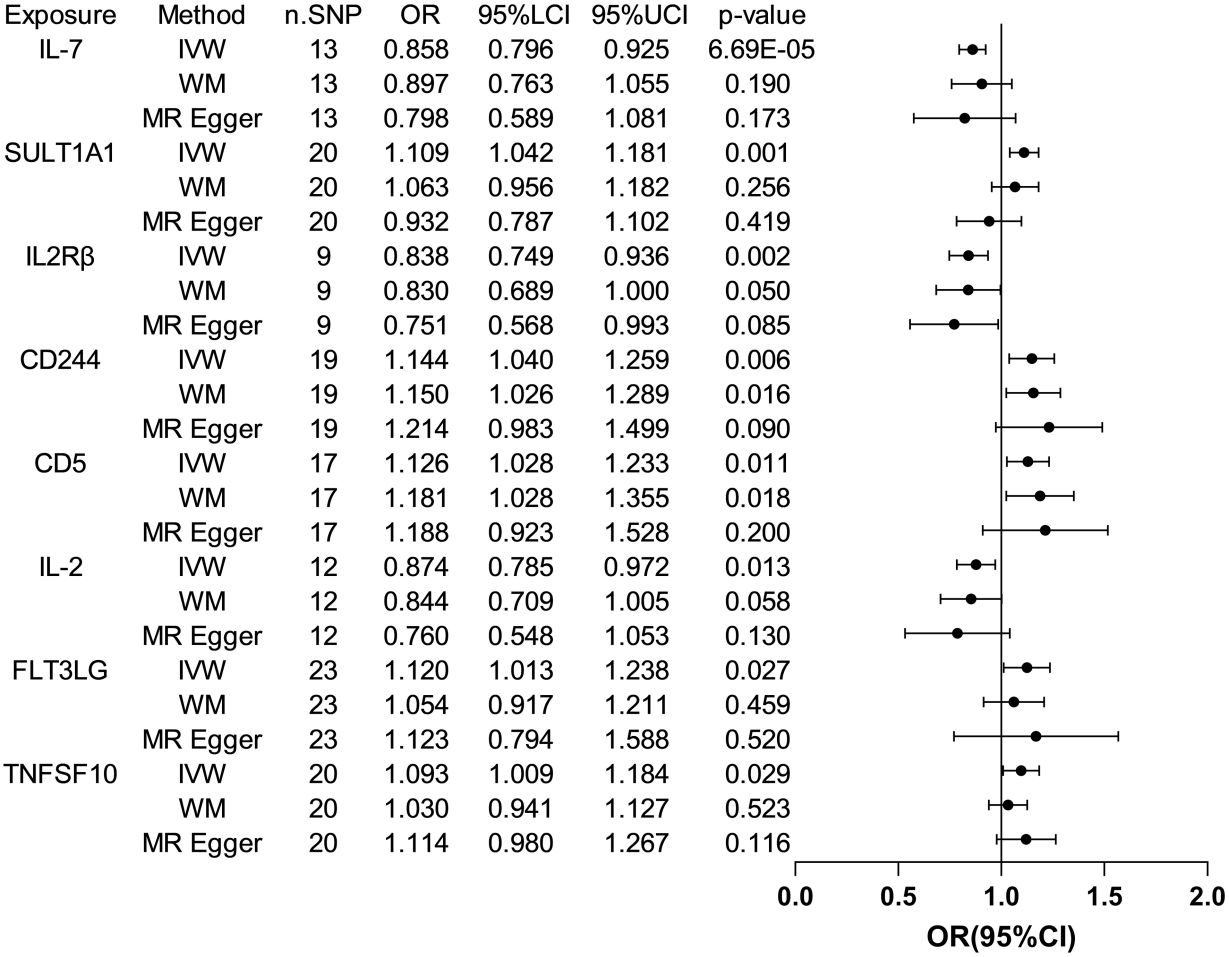
Forest plots of the pooled OR results between 8 inflammatory factors and ASD in the forward MR analysis.

**Figure 3:**
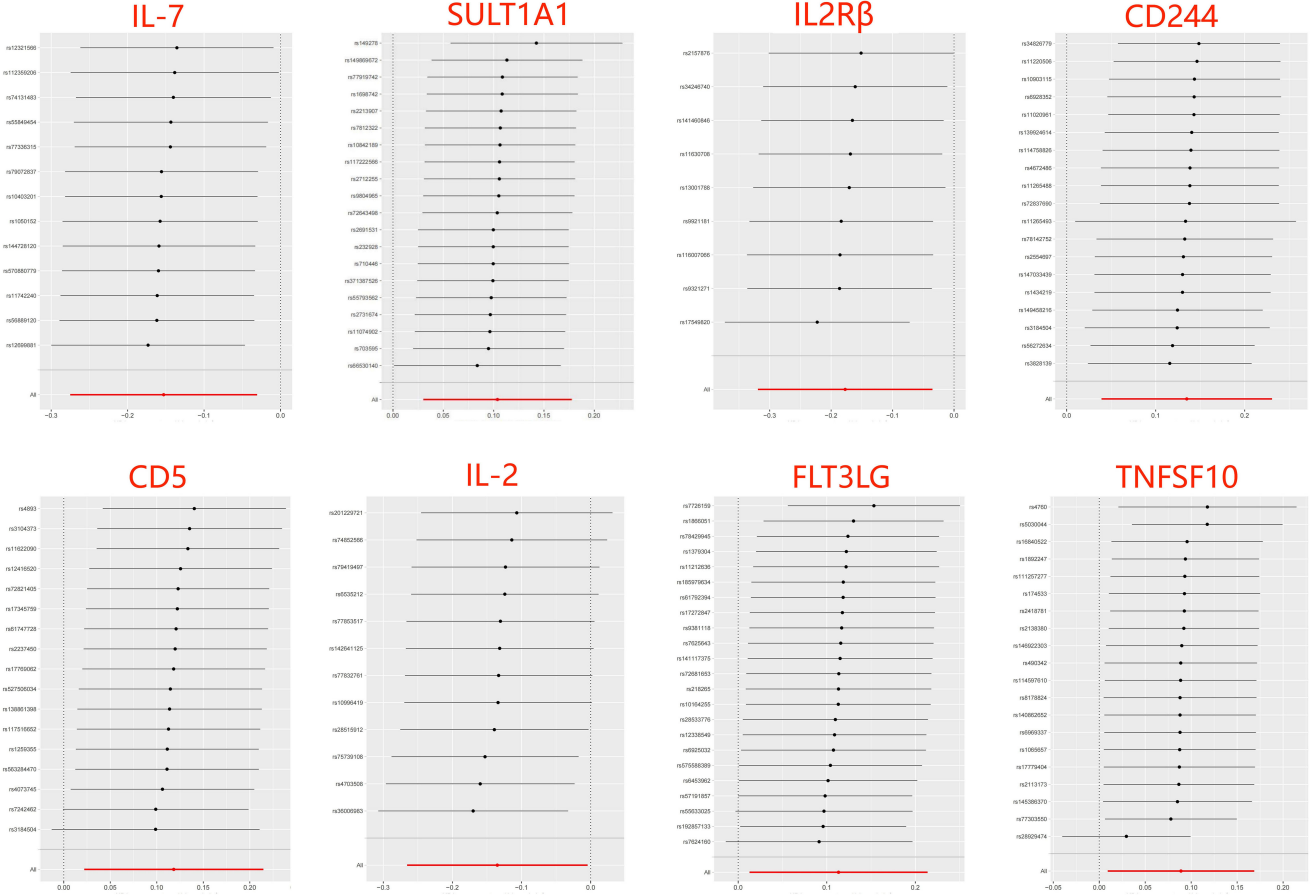
Leave-one-out of SNPs results between 8 inflammatory factors and ASD in the forward MR analysis. Each black point indicates the MR result of all remaining SNPs after removing the SNP in this line. The MR results estimated by all SNPs are depicted by the red point. No single SNP strongly drives the overall causal effect in these sensitivity analyses.

The analysis results from IVW showed a statistically significant negative correlation between levels of interleukin-7 (IL-7) and ASD (OR = 0.858, 95% CI = 0.796 to 0.925, *p* = 6.69e-05). The results also showed possible positive associations between elevated levels of sulfotransferase 1A1 (SULT1A1) (OR = 1.109, 95% CI = 1.0423 to 1.181, *p* = 0.001), natural killer cell receptor 2B4 (CD244) (OR = 1.144, 95% CI = 1.040 to 1.259, *p* = 0.006), T-cell surface glycoprotein CD5 (CD5) (OR = 1.126, 95% CI = 1.028 to 1.233, *p* = 0.011), Fms-related tyrosine kinase 3 ligand (FLT3LG) (OR = 1.120, 95% CI = 1.013 to 1.238, *p* = 0.027), and tumor necrosis factor-related apoptosis-inducing ligand (TNFSF10) (OR = 1.093 95% CI = 1.009 to 1.184, *p* = 0.029) and an increased occurrence of ASD. Levels of interleukin-2 receptor subunit beta (IL2Rβ) (OR = 0.838, 95% CI = 0.749 to 0.936, *p* = 0.002), and interleukin-2 (IL-2) (OR = 0.874, 95% CI = 0.785 to 0.972, *p* = 0.013) were inversely associated with the risk of ASD. The results of the WM analysis for CD244 (OR = 1.150, 95% CI = 1.026 to 1.289, *p* = 0.016) and CD5 (OR = 1.181, 95% CI = 1.028 to 1.355, *p* = 0.018) also indicate a causal relationship with ASD, consistent with the trend observed in the IVW method. MR Egger analysis of all the inflammatory factors did not find any significant causal relationship with ASD. **Figure 2** provide the results of the IVW, WM, and MR Egger analysis. None of the intercepts in the MR-Egger regression analysis significantly deviated from the zero (*p* > 0.05), suggesting no horizontal pleiotropy. Heterogeneity was observed only in TNFSF10 with a Cochran’s Q-derived *p <*0.05, but the causal estimate was acceptable when utilizing the random-effects IVW method (**Table 1**). The MR leave-one-out sensitivity analysis indicated that sequentially excluding individual SNP did not significantly influence the results, and all the estimates of the error lines were on the same side (**Figure 3**). The results of the IVW, WM, and MR Egger mode for all circulating inflammatory factors on ASD are displayed in **Supplementary Table S5**.

**Table 1:** The heterogeneity and horizontal pleiotropy results of the 8 inflammatory factors and ASD in the forward MR analysis.

Exposure	Heterogeneity test						Pleiotropy test		
	MR Egger			IVW			MR Egger		
	Q-value	Q-df	p-value	Q-value	Q-df	p-value	Intercept	SE	p-value
IL-7	4.28	11	0.961	4.54	12	0.971	0.0072	0.014	0.616
SULT1A1	8.55	18	0.969	13.68	19	0.802	0.0201	0.010	0.061
IL2Rβ	4.14	7	0.763	4.93	8	0.765	0.0130	0.015	0.405
CD244	25.00	17	0.094	25.60	18	0.109	-0.0066	0.011	0.546
CD5	14.10	15	0.521	14.30	16	0.579	-0.0056	0.012	0.657
IL-2	6.56	10	0.766	7.40	11	0.766	0.0165	0.018	0.382
FLT3LG	29.10	21	0.111	29.10	22	0.142	-0.0002	0.015	0.989
TNFSF10	34.9	18	0.010	35.5	19	0.013	-0.0042	0.011	0.708

MR, Mendelian randomization; Q, heterogeneity statistic Q; df, degree of freedom; SE, standard error.

### 3.3 The causative impact of ASD on circulating inflammatory factors

**Figure 4:**
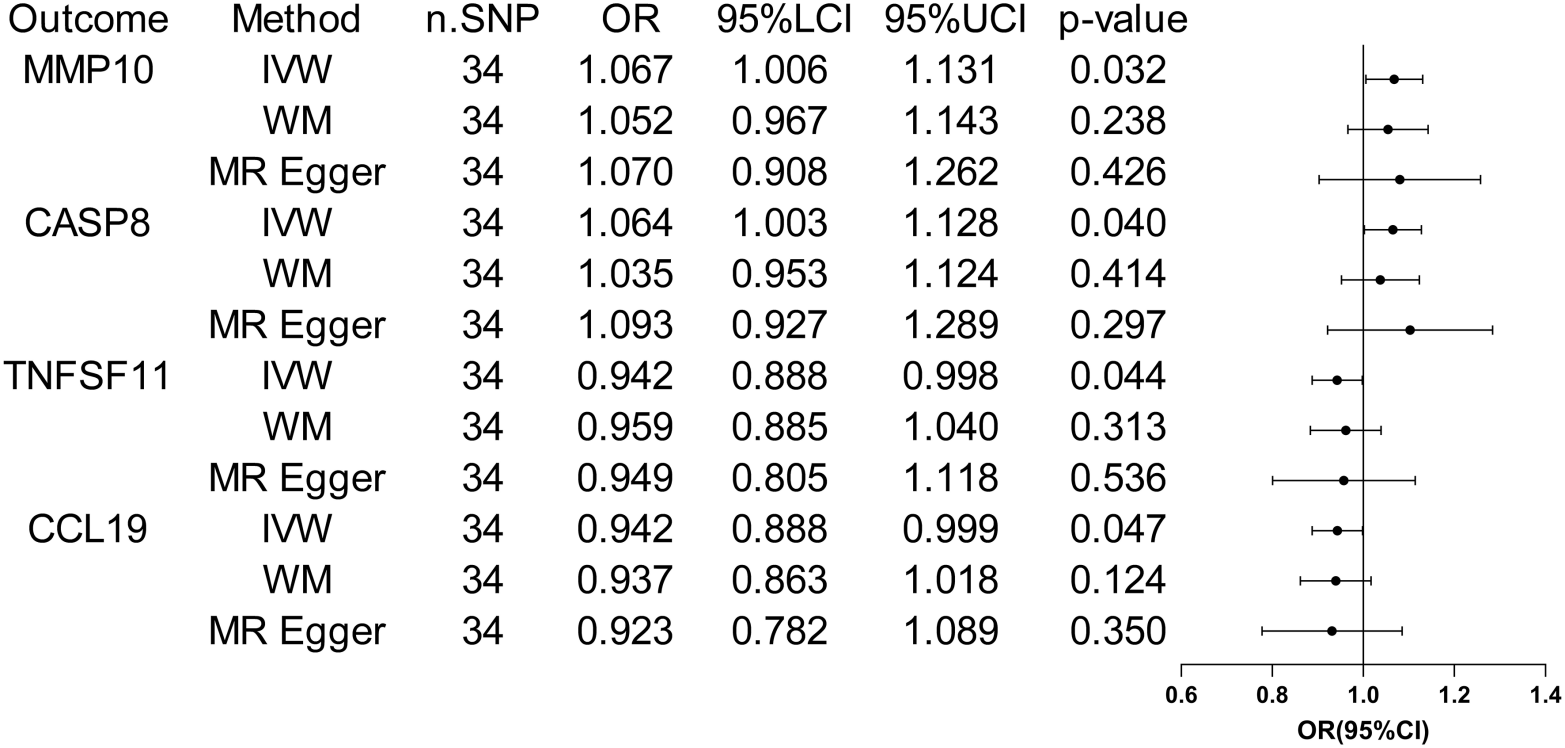
Forest plots of the pooled OR results between ASD and 4 inflammatory factors in the reverse MR analysis.

**Figure 5:**
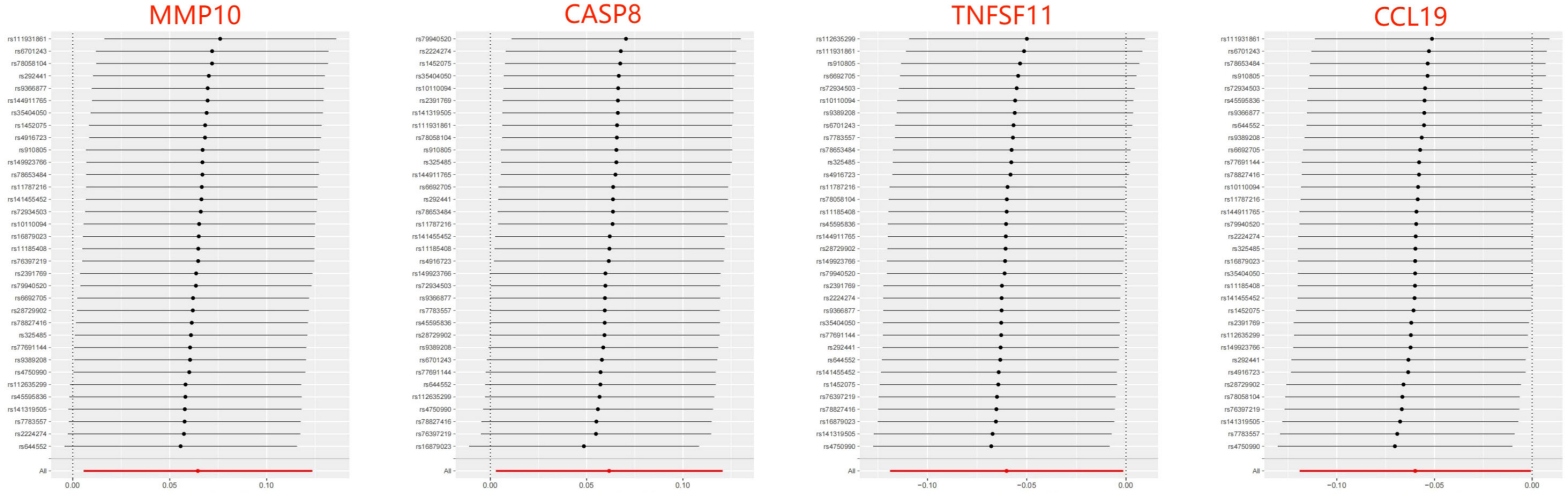
Leave-one-out of SNPs results between ASD and 4 inflammatory factors in the reverse MR analysis. Each black point indicates the MR result of all remaining SNPs after removing the SNP in this line. The MR results estimated by all SNPs are depicted by the red point. No single SNP strongly drives the overall causal effect in these sensitivity analyses.

When considering functional outcomes of ASD as exposures and the 91 circulating inflammatory factors as outcomes, the IVW results indicate that adverse functional outcomes following ASD may lead to increased levels of matrix metalloproteinase-10 (MMP10) and caspase 8 (CASP8) (OR = 1.067, 95% CI = 1.006 to 1.131, *p* = 0.032; OR = 1.064, 95% CI = 1.003-1.128, *p* = 0.040), as well as decreased levels of tumor necrosis factor-related activation-induced cytokine (TNFSF11) and C-C motif chemokine 19 (CCL19) (OR = 0.942, 95% CI = 0.888 to 0.998, *p* = 0.044; OR = 0.942 95% CI = 0.888 to 0.999, *p* = 0.047). The analysis results from both WM and MR Egger did not reveal any significant causality between ASD and the four inflammatory factors. **Figure 4** provide the results of the IVW, WM, and MR Egger analysis. Cochran’s Q-test results showed no evidence of heterogeneity in the causal relationship between these SNPs. The p-value of the MR-Egger intercept was greater than 0.05, indicating that horizontal pleiotropy was not possible for these four associations (**Table 2**). Furthermore, the sensitivity analysis proved the robustness of these observed causal associations (**Figure 5**). The results of the IVW, WM, and MR Egger mode for ASD on all circulating inflammatory factors are displayed in **Supplementary Table S6**.

**Table 2:** The heterogeneity and horizontal pleiotropy results of the ASD and 4 inflammatory factors in the reverse MR analysis.

Outcome	Heterogeneity test						Pleiotropy test		
	MR Egger			IVW			MR Egger		
	Q-value	Q-df	p-value	Q-value	Q-df	p-value	Intercept	SE	p-value
MMP10	28.34	32	0.652	28.34	33	0.698	-0.0003	0.008	0.965
CASP8	27.00	32	0.718	27.12	33	0.754	-0.0007	0.007	0.922
TNFSF11	24.04	32	0.843	24.05	33	0.872	-0.0002	0.015	0.989
CCL19	25.78	32	0.773	25.84	33	0.808	0.0020	0.0076	0.798

MR, Mendelian randomization; Q, heterogeneity statistic Q; df, degree of freedom; SE, standard error.

## 4 Discussion

Although recent observational studies have suggested an association between inflammatory factors and ASD (27–29), it is important to note that these results may be influenced by confounding factors, and the causal relationship between the two remains inconclusive. Using novel data and approaches, this bidirectional MR study offers a genetic insight into the potential causal relationship between circulating inflammatory factors and ASD. The findings of this study suggest that levels of SULT1A1, CD244, CD5, FLT3LG, and TNFSF10 are positively associated with the risk of ASD, while levels of IL-7, IL2Rβ, and IL-2 are inversely associated with the risk of ASD. Furthermore, the genetic susceptibility to ASD exhibited suggestive evidence of increased levels of MMP10 and CASP8, and decreased levels of TNFSF11 and CCL19. Importantly, sensitivity analyses supported the robustness of these results.

Individuals with ASD show alterations in circulating inflammatory factors along with abnormal peripheral blood levels of lymphocytes and macrophages across the lifespan (30). Inflammatory factor profiling can reveal the progression and status of immune system dysregulation in ASD, offering therapeutic targets for improving core autistic symptoms. Current results in the forward MR analysis demonstrated a strong causal association between a decreased level of circulating IL-7 and a higher liability of ASD based on the Bonferroni correction. IL-7 is mainly produced by stromal cells in lymphoid tissues and has pleiotropic effects on the development of T and B cells, as well as T-cell homeostasis. The administration or neutralization of IL-7 may enable the modulation of immune function in individuals with lymphocyte depletion or autoimmunity (31). Data from several studies have shown that plasma levels of IL-7 were higher in children with ASD than those observed in typically developing controls, but these differences did not reach statistical significance after correction for multiple comparisons (32–34). By contrast, Napolioni and colleagues (35) found that plasma levels of IL-7 were inversely correlated with full intelligence quotient in children with ASD using Spearman’s rank correlation analysis. Similarly, a large observational study on the risk of psychopathology found that decreased IL-7 levels in cord serum were linked to emotional symptoms and abnormal pro-social behavior in 5-year-old children (36). Overall, this MR analysis based on large populations replicates and extends these findings, highlighting a causal protective role of genetically encoded higher IL-7 levels against ASD.

Other results did not show robust causality after Bonferroni correction, which can only be regarded as suggestive evidence of potential causality. IL-2 functions as an essential immunoregulatory factor produced primarily by T cells, exerting its effects via binding to the high-affinity IL-2R comprising of α (IL-2Rα), β (IL-2Rβ), and γ (IL-2Rγ) subunits. Both IL-2 and IL-2Rβ have been implicated in clonal expansion and functional differentiation of T cells and natural killer cells (37, 38). Vojdani et al. have found that children with ASD appeared to suffer from decreased blood natural killer cell activity due to their low intracellular IL-2 levels (39). In patients with ASD, the proportion of DR+ (activated) T lymphocytes is abnormally increased, whereas the proportion of IL-2 receptor+ lymphocytes remains unchanged or even decreases. This is inversely proportional to the severity of autistic symptoms and similar to that seen in autoimmune diseases (40–42). In addition, previous studies have observed lower mRNA expression levels of IL-2 and percentages of IL-2 synthesizing CD4+ and CD8+ T cells in the peripheral blood of ASD children as compared to controls (43, 44). To sum up, our findings further support these observations and provide evidence of immune dysfunction and autoimmunity in patients with ASD.

Preliminary results suggest that there may be a positive association between levels of SULT1A1, CD244, CD5, FLT3LG, and TNFSF10 and risk of ASD. However, much of the research up to now has not dealt with the relationship between these inflammatory factors and ASD. SULT1A1 is responsible for the sulfonation of xenobiotics and has been implicated in several cancers by activating carcinogens (45). During autoimmune neuroinflammation, SULT1A is highly expressed in astrocytes, hindering the anti-inflammatory activity of endogenous estrogens (46). CD244 is an immune regulation receptor presented in all NK cells, which can stimulate NK cell cytotoxicity and IFN-γ production by interacting with CD48 on neighboring lymphocytes (47, 48). It has previously been observed that children with ASD had significantly higher serum and plasma levels of CD5 than those of normal controls, which is positively correlated with Childhood Autism Rating Scale score (49, 50). As a pan T cell marker, CD5 is highly expressed in a variety of autoimmune diseases, and this MR study provides new evidence that elevated levels of circulating CD5 may directly promote the development of ASD. In premature infants following respiratory viral infections, hyperoxia-induced high FLT3LG expression can lead to expansion and activation of lung CD103+ dendritic cells. The FLT3LG level is positively correlated with the level of proinflammatory cytokines (51). In addition, chronic HIV-1 patients also displayed significantly high levels of FLT3LG expression (52). TNFSF10 a proapoptotic member of the tumor necrosis factor family, has been shown to be highly up-regulated in patients with inflammatory bowel disease (53) and neurodegenerative diseases (54). The previous findings suggest a positive outcome for our MR results, indicating the need for further research on the current topic.

The occurrence of ASD may increase the levels of MMP10 and CASP8 and decrease the levels of TNFSF11 and CCL19 in the results of reverse MR analyses, suggesting that they may act as downstream factors in ASD. A recent proteomics analysis on plasma inflammation-related protein changes found that MMP-10 expression was significantly up-regulated in the ASD group compared with healthy children (29). Mild cognitive impairment individuals with elevated cerebrospinal fluid levels of MMP-10 had a higher likelihood of progression to Alzheimer’s type dementia and faster cognitive decline (55). CASP8 is a protease with both pro-death and pro-survival functions by mediating extrinsic apoptosis and suppressing necroptosis. Postmortem analysis results showed that the apoptosis was increased in the prefrontal cortex, hippocampus, and cerebellum of the autistic brain, as characterized by significantly increased levels of cleaved CASP8 (56). TNFSF11 exerts essential roles in lymph node organogenesis, cellular immunity, and osteoclastogenesis. For example, TNFSF11 can signal the augmentation of IFN-γ secretion and inhibit apoptosis of human monocyte-derived dendritic cells (57, 58). CCL19 has shown significant potential in the regulation of adaptive immune responses by coordinating dendritic cell migration and increasing interactions between dendritic cells, T cells, and B cells in secondary lymphoid tissues (59, 60). Hence, ASD-induced decreases in peripheral blood CCL19 and TNFSF11 levels may further exacerbate immune system disorders.

This MR study employed a large sample size and instrumental variables obtained from the GWAS database, ensuring statistical robustness in estimating causal associations and enhancing the credibility of results. By addressing the bias introduced by confounding factors and reverse causality through MR analysis, this study provides stronger evidence for assessing the causal relationship between inflammatory factor levels and the risk of ASD compared to traditional observational studies. Nevertheless, several limitations of the present study should be considered. Firstly, the present MR study can only provide statistical evidence for the causal association between circulating inflammatory factors and ASD, and further research is needed to investigate the potential mechanisms involved. Secondly, although current sensitivity analyses did not reveal any significant pleiotropy between SNPs, the effect of pleiotropy on the MR results cannot be completely ruled out. Thirdly, this MR study utilized pooled data from the GWAS database and did not analyze stratified risk factors related to ASD duration, severity, treatment, and comorbidities. Finally, the genetic data were mainly collected from individuals of European descent, and it is uncertain whether these findings are applicable to individuals of other ancestries.

## 5 Conclusion

Overall, this study provide novel ideas that IL-7, SULT1A1, IL2Rβ, CD244, CD5, IL-2, FLT3LG, and TNFSF10 may be upstream factors in the pathogenesis of ASD, while levels of MMP10, CASP8, TNFSF11, and CCL19 may act as downstream factors in ASD. These inflammatory factors could potentially serve as biomarkers for early diagnosis and treatment of ASD.

## Data availability statement

The original contributions presented in the study are included in the article/**Supplementary Material**. Further inquiries can be directed to the corresponding author.

## Ethics statement

Ethical approval was not required for the study involving humans in accordance with the local legislation and institutional requirements. Written informed consent to participate in this study was not required from the participants or the participants’ legal guardians/next of kin in accordance with the national legislation and the institutional requirements.

## Author contributions

JL: Writing – review & editing, Writing – original draft, Resources, Methodology, Investigation, Formal Analysis, Data curation, Conceptualization. HD: Writing – review & editing, Writing – original draft, Visualization, Software, Investigation, Formal Analysis. WS: Writing – review & editing, Visualization, Software, Methodology, Investigation. MM: Writing – review & editing, Validation, Resources, Methodology. HZ: Writing – review & editing, Writing – original draft, Supervision, Project administration, Investigation, Conceptualization.
